# The Effect of Racial Concordance on Patient Trust in Online Videos About Prostate Cancer: A Randomized Clinical Trial

**DOI:** 10.1001/jamanetworkopen.2023.24395

**Published:** 2023-07-19

**Authors:** Stacy Loeb, Joseph E. Ravenell, Scarlett Lin Gomez, Hala T. Borno, Katherine Siu, Tatiana Sanchez Nolasco, Nataliya Byrne, Godfrey Wilson, Derek M. Griffith, Rob Crocker, Robert Sherman, Samuel L. Washington, Aisha T. Langford

**Affiliations:** 1 Department of Urology, New York University Langone Health, New York; 2 Department of Population Health, New York University Langone Health, New York; 3 Department of Surgery/Urology, Manhattan Veterans Affairs, New York, New York; 4 Department of Urology and Epidemiology and Biostatistics, Helen Diller Family Comprehensive Cancer Center, University of California San Francisco; 5 Trial Library, Inc., San Francisco, California; 6 Stakeholder Advisory Board, New York, New York; 7 Georgetown University, Washington, DC

## Abstract

**Importance:**

Black men have a higher risk of prostate cancer compared with White men, but Black adults are underrepresented in online content about prostate cancer. Across racial groups, the internet is a popular source of health information; Black adults are more likely to trust online health information, yet have more medical mistrust than White adults.

**Objective:**

To evaluate the association between racial representation in online content about prostate cancer and trust in the content and identify factors that influence trust.

**Design, Setting, and Participants:**

A randomized clinical trial was conducted from August 18, 2021, to January 7, 2022, consisting of a 1-time online survey. Participants included US men and women aged 40 years and older. Data were analyzed from January 2022 to June 2023.

**Interventions:**

Participants were randomized to watch the same video script about either prostate cancer screening or clinical trials presented by 1 of 4 speakers: a Black physician, a Black patient, a White physician, or a White patient, followed by a questionnaire.

**Main Outcomes and Measures:**

The primary outcome was a published scale for trust in the information. χ^2^ tests and multivariable logistic regression were used to compare trust according to the video’s speaker and topic.

**Results:**

Among 2904 participants, 1801 (62%) were men, and the median (IQR) age was 59 (47-69) years. Among 1703 Black adults, a greater proportion had high trust in videos with Black speakers vs White speakers (72.7% vs 64.3%; adjusted odds ratio [aOR], 1.62; 95% CI, 1.28-2.05; *P* < .001); less trust with patient vs physician presenter (64.6% vs 72.5%; aOR, 0.63; 95% CI, 0.49-0.80; *P* < .001) and about clinical trials vs screening (66.3% vs 70.7%; aOR, 0.78; 95% CI, 0.62-0.99; *P* = .04). Among White adults, a lower proportion had high trust in videos featuring a patient vs physician (72.0% vs 78.6%; aOR, 0.71; 95% CI, 0.54-0.95; *P* = .02) and clinical trials vs screening (71.4% vs 79.1%; aOR, 0.57; 95% CI, 0.42-0.76; *P* < .001), but no difference for Black vs White presenters (76.8% vs 73.7%; aOR, 1.11; 95% CI, 0.83-1.48; *P* = .49).

**Conclusions and Relevance:**

In this randomized clinical trial, prostate cancer information was considered more trustworthy when delivered by a physician, but racial concordance was significantly associated with trust only among Black adults. These results highlight the importance of physician participation and increasing racial diversity in public dissemination of health information and an ongoing need for public education about clinical trials.

**Trial Registration:**

ClinicalTrials.gov Identifier: NCT05886751

## Key Points

**Question:**

Are the race of the speaker, qualifications of the speaker, or topic of information associated with trust in online video content about prostate cancer?

**Findings:**

In this randomized clinical trial of 2904 US men and women, Black adults were significantly more likely to trust videos with a Black vs a White presenter, whereas among White adults, there was no significant association between trust and race of the presenter.

**Meaning:**

In contexts such as Black men’s disproportionate mortality from prostate cancer, racial representation may be an important tool to improve trust in populations who need the information most.

## Teaser

This randomized clinical trial evaluates the effect of racial concordance, speaker qualifications, and topic on patient trust in online videos about prostate cancer.

## Introduction

Prostate cancer is the second leading cause of cancer-related death among US men. Black men are significantly more likely to receive a prostate cancer diagnosis, have worse quality of life, and are more likely to die from prostate cancer compared with non-Hispanic White men.^1^ A recent meta-analysis suggested a significant interaction between race and social determinants of health with respect to prostate cancer-specific mortality.^2^ In addition, Black men are significantly underrepresented in prostate cancer clinical trials.^3^

Black adults have significantly more medical mistrust than their White counterparts.^4^ Although mistrust is often framed as a characteristic of individuals or communities such as Black adults, it is important to rethink and relocate mistrust as a phenomenon created by and existing within a system of structural racism and inequities in access to and quality of health care.^5,6^ For groups with a history of experiencing racism and other forms of discrimination, concerns about the trustworthiness of clinicians, health care institutions, and health care systems persist.^7^ The willingness to trust and act on information is shaped by assessments of the intent, character, motivations, and values of the source of health care information or health care services.^8^

Notably, Black adults are more likely to trust online health information compared with White adults.^9^ However, we reported that Black people are significantly underrepresented in online content about prostate cancer and clinical trials compared with US census data and prostate cancer patient demographics.^10,11^ It is unknown whether this influences decision-making about prostate cancer screening and clinical trial participation.

There is substantial literature that racial concordance with clinicians is positively associated with perceived quality of health care for Black adults.^12^ It is unknown whether racial concordance in online content impacts whether Black adults trust the material.

We conducted a randomized clinical trial of the impact of racial concordance and other source characteristics on health consumers’ trust (primary end point) of online health videos. We focused on prostate cancer screening and clinical trials given the substantial burden of disease and well-documented racial disparities. We hypothesized that Black men and women would have greater trust in videos delivered by a Black presenter, and that White adults’ trust would not be associated with presenter race. We also tested whether the speaker qualifications (patient vs physician) and topic (prostate cancer screening vs clinical trials) were associated with viewer trust.

## Methods

### Design of Intervention

The study team, including clinicians, behavioral scientists, and community stakeholders, collaboratively designed and filmed a video script about prostate cancer screening and a separate script about clinical trials with 4 different presenters (Black physician, White physician, Black patient, and White patient). All presenters were men. Videos were embedded into a Qualtrics survey including a baseline questionnaire with demographics (including participants’ self-reported gender and race and ethnicity), health literacy, eHealth Literacy Scale (eHEALS), and medical mistrust (Group-Based Medical Mistrust Scale). After the video, participants were asked whether they trusted the information using a published 4-point Likert scale of trust in clinicians.^13^ The study was approved by the NYU School of Medicine institutional review board. All prospective participants for the online study were presented with an initial webpage including IRB-approved information about the research study. Participants were asked to acknowledge that they reviewed the information and clicked to indicate their consent to enter into the Qualtrics survey. This study followed Preferred Reporting Items for Systematic Reviews and Meta-analyses (PRISMA) and American Association for Public Opinion Research (AAPOR) reporting guidelines. The trial protocol appears in Supplement 1. According to the NYU School of Medicine Office of Science and Research, the study did not meet criteria for a randomized clinical trial (ie, not a clinical trial of a drug, biological, or device; not an NIH-funded clinical trial; and not studying an intervention used to modify a biomedical or health-related outcome); therefore, it was not registered prospectively on clinicaltrials.gov. At the request of the journal editors, the study was retroactively registered on clinicaltrials.gov and the report follows the Consolidated Standards of Reporting Trials (CONSORT) reporting guideline.

### Recruitment

Eligibility criteria were Black or White US adults aged 40 years and older. Participants were recruited by Dynata, a large online survey platform used in prior studies.^14^ Participants were recruited from across the US and not from any specific health system. Although prostate cancer affects men, women were included because they seek prostate cancer information online and often play an important role in prostate cancer screening and treatment decisions.^15^ We planned a priori to recruit an approximately 4:1 ratio of men to women according to the demographics of prostate cancer information-seekers in a large online health community.^16^ Our prespecified plan was to recruit 1200 Black adults (900 men and 300 women) and 1200 White adults (900 men and 300 women). However, due to an initially robust response among Black women, we further stratified recruitment by gender using an upfront screening questionnaire to recruit additional Black men, and in the subsequent fielding of separate surveys for White men and women participants (see the eFigure in Supplement 2 for study flow according to PRISMA/AAPOR guidelines).

### Randomization

We conducted a stratified randomization of Black and White adults. After online consent by clicking assent on the entry page, participants were randomized in parallel in 8 blocks by the randomizer tool within Qualtrics to a survey containing 1 of the 8 videos. The surveys contained identical questions except for the embedded videos.

### Statistical Analysis

Data were collected within Qualtrics. Statistical analysis was performed using Stata version 17 SE (StataCorp) for data management and descriptive statistics, and the epiR and survival packages from R version 4.2.1 (R Project for Statistical Computing) to measure associations. Using the χ^2^ test, we assessed whether there were differences in trust (primary outcome) in videos with a Black vs White presenter, physician vs patient, and screening vs clinical trials topic. To provide conservative estimates and as in previous studies,^13^ our power calculation was performed with trust as a dichotomous variable (trust completely or most of the information vs only trust some or not at all) and assuming a 10% difference in proportions. A sample size of 1200 Black adults (or White adults) gives 92% power to detect a 10% difference in trust according to presenter race, qualifications, or video topic. Logistic regression was used for multivariable analysis to examine (1) video features associated with trust with mutual adjustment (speaker race, qualifications, or topic) and (2) individual factors associated with trust across all prostate cancer videos (ie, demographics, health literacy using a validated 3-item scale,^17^ eHealth literacy using eHEALS,^18^ and medical mistrust^19^ as well as video features). Statistical significance was considered as *P* < .05. Data were analyzed from January 2022 to June 2023.

## Results

Most participants were men (1801 participants [62%]), 236 (8%) had a history of prostate cancer, and 1703 (59%) were Black (Table 1; eTable 1 and eTable 2 in Supplement 2).^20^ Among Black adults, a greater proportion had high trust in videos with a Black speaker vs White (72.7% vs 64.3%) and physician vs patient (72.5% vs 64.6%), and a marginally higher proportion had high trust in videos about screening vs clinical trials (70.7% vs 66.3%). Among adults randomized to videos with a Black speaker, Black adults had significantly less trust in a Black patient vs a Black physician (OR, 0.68; 95% CI, 0.50-0.93; *P* = .01).

**Table 1.** Demographics of the Study Population Including 1703 Black Adults and 1201 White Adults Randomly Assigned to Watch 1 of 8 Videos About Prostate Cancer

Variable	Participants, No. (%)	
	Black adults (n = 1703)	White adults (n = 1201)
Age, Mean (SD) [range], y	55.5 (11.0) [40-91]	63.0 (11.8) [40-91]
Gender		
Women	802 (47.1)	301 (25.1)
Men	901 (52.9)	900 (74.9)
Ethnicity^a^		
Non-Hispanic	1582 (92.9)	1195 (99.5)
Hispanic	115 (6.8)	3 (0.3)
Education		
Less than high school	26 (1.5)	19 (1.6)
High school graduate	328 (19.3)	203 (16.9)
Some college	479 (28.1)	315 (26.2)
College graduate	548 (32.2)	426 (35.5)
Graduate/professional degree	314 (18.4)	236 (19.7)
Other	8 (0.5)	2 (0.2)
Personal cancer history		
No cancer history	1464 (86.0)	1010 (84.1)
Prostate cancer	155 (9.1)	81 (6.7)
Any other cancer	84 (4.9)	110 (9.2)
Health literacy^b^		
Mean (SD) [range]	2.4 (2.6) [0-12]	2.1 (2.3) [0-12]
Adequate health literacy	519 (30.5)	398 (33.14)
Limited health literacy	1184 (69.5)	803 (66.9)
eHEALS^c^		
Mean (SD) [range]	31.8 (5.7) [8-40]	30.9 (5.8) [8-40]
Group-Based Medical Mistrust Scale^d^		
Mean (SD) [range]	13.9 (6.3) [6-30]	9.8 (4.8) [6-30]

Abbreviation: eHEALS, eHealth Literacy Scale.

^a^ Six Black adults and 3 White adults did not know or wish to answer their ethnicity.

^b^ Health literacy was categorized into adequate and limited, as in Wynia et al.^20^

^c^ Higher scores on eHEALS indicate greater eHealth literacy. A total of 249 Black adults and 97 White adults skipped at least 1 question on the eHEALS questionnaire.

^d^ Higher scores indicate greater medical mistrust.

Among White adults, a greater proportion had high trust in videos featuring a physician vs patient (78.6% vs 72.0%) and about screening vs clinical trials (79.1% vs 71.4%). However, there was no difference for a Black vs White presenter (76.8% vs 73.7%).

On multivariable analysis mutually adjusted for the type of video (Table 2), among Black adults, trust was significantly higher with a Black vs White presenter and lower with a patient vs physician and clinical trials vs screening. As shown in eTable 3 in Supplement 2, when stratified by health literacy, Black adults had significantly more trust in videos with a Black vs White presenter, and lower with a patient vs a physician, in both the limited and adequate health literacy strata. Among White adults, there was significantly lower trust in videos with a patient vs physician, and clinical trials vs screening. There was no difference in trust by presenter race.

**Table 2.** Multivariable Logistic Regression Model for Trust in Prostate Cancer Videos According to Video Characteristics (Race of Speaker, Qualifications of Speaker, and Topic of the Video), Mutually Adjusted

Characteristic	Black adults (n = 1703)		White adults (n = 1201)	
	Adjusted OR (95% CI)	*P* value	Adjusted OR (95% CI)	*P* value
Black vs White speaker	1.48 (1.21-1.82)	.001	1.18 (0.91-1.54)	.21
Patient vs physician	0.69 (0.56-0.85)	.001	0.70 (0.54-0.91)	.01
Clinical trials vs screening	0.81 (0.66-0.99)	.04	0.66 (0.51-0.86)	.002

Abbreviation: OR, odds ratio.

Additional logistic regression models examined individual factors associated with trust in prostate cancer information across all videos (Table 3). For Black adults, Black speaker (aOR, 1.62; 95% CI, 1.28-2.05; *P* < 0.001) had a significant positive association with trust, whereas patient speaker (aOR, 0.63; 95% CI, 0.49-0.80; *P* < 0.001) and clinical trial topic (aOR, 0.78; 95% CI, 0.62-0.99; *P* = 0.04) were associated with a lower odds of trust in the video. Gender, history of prostate cancer, health literacy, e-Health literacy, and medical mistrust were also associated with trust in the video among Black adults. For White adults, patient presenter (aOR, 0.71; 95% CI, 0.54-0.95; *P* = 0.02) and clinical trial topic (aOR, 0.57; 95% CI, 0.42-0.76; *P* < 0.001) were associated with a significantly lower odds of trust, whereas there was no significant difference according to race of the presenter (aOR, 1.11; 95% CI, 0.83-1.48; *P* = 0.49). Other factors associated with trust in the video among White adults included a history of prostate cancer, e-Health literacy, and medical mistrust.

**Table 3.** Univariable and Multivariable Logistic Regression Analysis of Individual Factors From the Questionnaire Associated With Trust Across All Prostate Cancer Videos

Factor	Black adults				White adults			
	Univariable regression		Multivariable regression		Univariable regression		Multivariable regression	
	OR (95% CI)	*P* value	Adjusted OR (95% CI)	*P* value	OR (95% CI)	*P* value	Adjusted OR (95% CI)	*P* value
Race of speaker								
White	1 [Reference]	NA	1 [Reference]	NA	1 [Reference]	NA	1 [Reference]	NA
Black	1.48 (1.21-1.82)	<.001	1.62 (1.28-2.05)	<.001	1.18 (0.91-1.54)	.21	1.11 (0.83-1.48)	.49
Type of speaker								
Physician	1 [Reference]	NA	1 [Reference]	NA	1 [Reference]	NA	1 [Reference]	NA
Patient	0.69 (0.56-0.85)	<.001	0.63 (0.49-0.80)	<.001	0.70 (0.54-0.91)	.01^a^	0.71 (0.54-0.95)	.02
Topic of video								
Screening	1 [Reference]	NA	1 [Reference]	NA	1 [Reference]	NA	1 [Reference]	NA
Clinical trial	0.82 (0.67-1.00)	.06	0.78 (0.62-0.99)	.04	0.66 (0.51-0.86)	.002^a^	0.57 (0.42-0.76)	<.001
Age (decade)	1.11 (1.01-1.23)	.03	1.09 (0.97-1.23)	.15	1.04 (0.93-1.17)	.45	1.00 (0.88-1.14)	.98
Gender								
Women	1 [Reference]	NA	1 [Reference]	NA	1 [Reference]	NA	1 [Reference]	NA
Men	1.46 (1.19-1.79)	<.001	1.34 (1.05-1.70)	.02	1.25 (0.93-1.68)	.14	1.13 (0.81-1.59)	.48
Ethnicity								
Non-Hispanic	1 [Reference]	NA	1 [Reference]	NA	1 [Reference]	NA	1 [Reference]	NA
Hispanic	1.05 (0.70-1.58)	.82	0.92 (0.49-1.73)	.79	0.65 (0.06-7.22)	.73	0.60 (0.05-7.30)	.69
College degree								
No college degree	1 [Reference]	NA	1 [Reference]	NA	1 [Reference]	NA	1 [Reference]	NA
Earned college degree or above	1.36 (1.11-1.67)	.003	1.03 (0.82-1.31)	.78	1.28 (0.98-1.66)	.07	1.14 (0.85-1.54)	.38
Personal cancer history								
No prostate cancer or any other cancer history	1 [Reference]	NA	1 [Reference]	NA	1 [Reference]	NA	1 [Reference]	NA
Prostate cancer history	1.74 (1.17-2.59)	.006	1.79 (1.03-3.14)	.04	2.22 (1.16-4.25)	.02	2.29 (1.12-4.67)	.02
Any other cancer history	1.47 (0.89-2.43)	.14	1.71 (0.95-3.08)	.07	1.18 (0.74-1.89)	.48	1.12 (0.67-1.87)	.65
Health literacy								
Adequate health literacy	1 [Reference]	NA	1 [Reference]	NA	1 [Reference]	NA	1 [Reference]	NA
Limited health literacy	0.51 (0.40-0.65)	<.001	0.69 (0.53-0.91)	.01	0.57 (0.42-0.76)	<.001^a^	0.78 (0.56-1.10)	.15
eHealth literacy^a^	1.06 (1.04-1.08)	<.001	1.06 (1.04-1.08)	<.001	1.05 (1.03-1.08)	<.001	1.05 (1.02-1.08)	<.001
Group-Based Medical Mistrust Scale^a^	0.95 (0.94-0.97)	<.001	0.94 (0.92-0.96)	<.001	0.93 (0.91-0.96)	<.001	0.93 (0.90-0.96)	<.001

Abbreviations: NA, not applicable; OR, odds ratio.

^a^ eHealth literacy (measured with eHealth Literacy Scale, which has 8 questions with scores ranging from 1-5 resulting in a final score of 8-40 which was modeled as a continuous variable, with higher scores indicating greater eHealth literacy) and medical mistrust (measured with the Group-Based Medical Mistrust Scale with 6 items scored from 1 = strongly disagree to 5 = strongly agree, resulting in a final score ranging from 6-30 which was modeled as a continous variable with higher scores indicating greater mistrust).

## Discussion

In this randomized clinical trial of online prostate cancer videos, for both Black and White adults, physicians were trusted more than patients as the source of information, and there was less trust in videos about clinical trials vs screening. Black adults had significantly greater trust in information presented by a Black speaker.

These results are consistent with other studies highlighting the importance of racial concordance in health information for Black adults. For example, in a randomized clinical trial of physician-delivered videos about COVID-19, messages from race and ethnicity–concordant (vs discordant) physicians significantly increased information-seeking among Black participants.^21^

Our results highlight actionable opportunities to improve care. They suggest the importance of increasing diversity in medical information for patients, and the importance of physicians as trusted sources. Particularly considering the substantial amount of circulating misinformation,^22^ it is essential for physicians to actively disseminate evidence-based content online.

Our data also underscore the importance of public education about the value of clinical research and protection of human participants. Black adults represent only 0.5% and 6.7% of participants in prostate cancer screening and treatment trials, respectively,^23^ which has critical implications for the relevance of how research data are used to inform clinical care and health policy.

Online platforms are an important source of health information, yet content on these platforms is not representative of diverse populations.^10,11^ We found that representation matters. For health conditions with observed racial disparities such as prostate cancer, messages delivered from racially concordant presenters, particularly physicians, are important to increase trust. Thus, it may be beneficial to tailor health communications by perceived racial group, specifically in instances such as prostate cancer where a specific group (eg, Black men) has consistently worse health outcomes and has sought care in a historical context for mistrust, such as structural racism.^24^ Increasing the representation of physicians (or actors) who appear concordant with the target audience may be an important tool for more effective health communication strategies to mitigate health disparities.

### Limitations and Strengths

Limitations of our study are that a small proportion of surveys were excluded that failed quality control (Recapcha), and representativeness of online survey takers is unknown. Our findings may not be generalizable to people who do not use the internet. However, since our focus is the impact of online communications on health disparities, the primary target audience is internet users. Additionally, all the video presenters were men and the clinicians were physicians, so we cannot assess the impact of gender, or trust in other types of health professionals (eg, nurses). Furthermore, we did not collect data on social determinants of health or geography, so we are unable to determine whether trust varied by these factors. Strengths of our study include it being the first randomized trial to our knowledge comparing trust in health-related videos according to the presenter’s race and qualifications.

## Conclusions

Key findings are that racial representation in online sources matters. Also, physicians are essential contributors to online health content, and increasing public trust in clinical trials is critical.

## Notes

Supplement 1.
Trial Protocol

Supplement 2. **eTable 1. **Demographics for Black Participants Who Were Randomized to Each of 8 Different Videos **eTable 2. **Demographics for White Participants Who Were Randomized to Each of 8 Different Videos **eTable 3. **Multivariable Logistic Regression Model for Trust in Prostate Cancer Videos According to Video Characteristics (Race of Speaker, Qualifications of Speaker and Topic of the Video), Mutually Adjusted, Among 1703 Black Adults Stratified by Health Literacy (Limited or Adequate) **eFigure. **Flow Chart for Selection of the Study Population

Supplement 3.
Data Sharing Statement
