# Supplementary material for: The Effect of Racial Concordance on Patient Trust in Online Videos About Prostate Cancer: A Randomized Clinical Trial
Source: JAMA Netw Open. 2023 Jul 19;6(7):e2324395. doi: 10.1001/jamanetworkopen.2023.24395 (PMC10357333; doi:10.1001/jamanetworkopen.2023.24395)
Supplement: Supplement 1. — Trial Protocol [file jamanetwopen-e2324395-s001.pdf]

Supplement. Study Protocol

**Brief Summary**	This study is part of a grant from the DoD to study the impact of online information on health disparities. The first step is a randomized trial examining trust in online prostate cancer videos among U.S. adults based on the race and qualifications of the speaker.
**Phase**	N/A
**Objectives**	The primary objective is to examine the impact of source characteristics on health consumers' trust in online videos.
**Methodology**	A randomized trial where health consumers are randomized to watch a video by one of 4 speakers about prostate cancer.
**Primary Endpoint**	The primary endpoint is trust in the video.
**Study Duration**	12 months
**Participant Duration**	Participants will watch a video and complete a questionnaire (approximately 15 minutes total).
**Duration of IP administration**	N/A
**Population**	U.S. adults over age 40
**Study Sites**	Online study coordinated by NYU
**Description of Study Agent/Procedure**	The intervention is viewing an online video
**Reference Therapy**	N/A
**Key Procedures**	N/A
**Statistical Analysis**	Chi-square tests will be used to assess the relationship between trust (and other secondary outcome measures) between videos featuring 1) physician/non-physician speaker and 2) Black/White speaker, 3) Screening/Clinical Trials topic.

Name and Description of the Investigational Agent

With oversight from co-investigators and input from the stakeholder advisory board for this grant, we will design a video script about prostate cancer (PCa) screening and clinical trials. We will work with expert videographers from the NYU Media Team to film the scripts with 4 different presenters (Black physician, White physician, Black patient, White patient). Consenting

participants from the Dynata online panel (formerly known as Survey Sampling International (SSI)) will be randomly assigned to view a video by one of 4 speakers. The intervention is meant to be completed at home or anywhere else they choose (i.e., not as part of a medical encounter).

Preclinical Data

N/A

Clinical Data to Date

Our group has previously published on the quality of YouTube videos about prostate cancer, and engagement of YouTube users with these videos. Since this trial has not yet begun, there is currently no available clinical research data to directly compare the response of users to the same video script with different narrators.

Dose Rationale

N/A

Rationale

Black adults have significantly more medical mistrust than other racial/ethnic groups. There is also substantial literature documenting that racial concordance positively influences perceived quality of healthcare for Black adults, and that in general, Black adults are more likely to trust online health information. It is unknown whether the source and representativeness of online PCa content impacts how much Black adults trust the material.

Our hypotheses are as follows:

- Black adults are more likely to trust online PCa content that is racially concordant than content that lacks Black representation.

- eHealth literacy and medical mistrust are significantly associated with trust in online PCa content.

Based on feedback from the scientific reviewers from the DoD grant proposal, a parallel group of White adults was added.

Known Potential Risks

Potential risks include feeling upset as a result of watching the videos or completing the questionnaires, or misunderstanding of the information about PCa; however, participants will be told that they can exit at any time. The risk of a breach in confidentiality is unlikely since two separate platforms are being used for recruitment and data collection.

These minor risks are outweighed by the value of information to be gained from these studies in terms of improving the provision of information to Black men and potentially reducing health disparities through improved communication.

Known Potential Benefits

The potential benefit of this grant-funded study is to examine the trust of Black adults in different types of online information about prostate cancer. Results will be disseminated widely through the media, consumer health libraries and partnerships with key stakeholders, and can immediately be used to inform online health communications about PCa for Black men. The long-range potential benefits include reducing health disparities in PCa survival and quality of life through an improvement in online information provision about PCa.

Primary Objective

To examine trust of online PCa information among Black adults through an experimental study.

Study Design and Endpoints

We will perform a nationwide randomized trial (phase N/A) where U.S. adult participants of an online survey panel are randomized to view a video by one of 4 speakers about prostate cancer and complete a questionnaire.

The primary endpoint is trust in the video using a published Likert scale, which addresses our objective of evaluating predictors of trust in PCa online content. This endpoint was chosen due to previous data suggesting that racial concordance is an important factor in trust during physician encounters, but has not been studied in the context of PCa online videos.

Inclusion Criteria

Phase 1 (Quantitative): In order to be eligible to participate in this study, an individual must meet all of the following criteria:

1. U.S. adult age 40+
2. Enrolled in Dynata online survey panel

Exclusion Criteria

An individual who meets any of the following criteria will be excluded from participation in this study:

1. Children
2. Individuals living outside the U.S.

Vulnerable Subjects

N/A

Strategies for Recruitment and Retention

We will recruit Black and White adults using the nationwide platform Dynata. Only individuals completing the full survey will be compensated and count toward the study, and only individuals whose survey passes the quality control checks will be included in data analysis. Any individuals who do not complete the survey will be replaced by Dynata until the enrollment target is reached.

Upon receipt of IRB approval, we will use Dynata to recruit Black adults and White adults (regardless of ethnicity). The recruitment messages will be sent to at least 5000 adults, until at least 1200 surveys each are fully completed by eligible Black and White adults (including at least 900 men) that pass quality control to fulfill our power calculation. Participants will be randomized to view a video with one of the 4 speakers through random computer assignment.

All prospective participants for the online study will be presented with an initial webpage including IRB-approved information about the research study and that they can exit at any time. The page will also contain a disclaimer that the videos should not be used as medical advice. Participants will be asked to acknowledge that they have reviewed this information and click to indicate their consent to enter into the Qualtrics survey. After completion of the survey, respondents will receive their standard incentive from Dynata. The study team will perform quality checks on the data to determine the RECAPCHA score and response to attention questions to identify suspicious responses, which will be excluded from the analytic sample. The study team will aggregate data from the remaining surveys that pass quality control checks and descriptive statistics will be calculated. Only de-identified data will be used in publications about the trial, and no personal identifiable information will be included. Retention of participants is not required beyond completion of the survey. The trial poses no more than minimal risk to subjects. Potential risks include feeling upset as a result of watching the videos or completing the questionnaires, or misunderstanding of the information about PCa. The risk of a breach in confidentiality is unlikely since two separate platforms are being used for recruitment and data collection. Participants will be recruited and paid through Dynata. However, the survey will be programmed and housed in Qualtrics. Therefore Dynata does not have access to the survey data, and conversely the study team cannot link the responses back to individual online survey-takers. Thus, the risk of a breach in confidentiality or leak of personal information is minimal.

Duration of Study Participation

The expected duration of participation is approximately 15 minutes. Participants will be asked to watch a video and complete questions.

Total Number of Participants and Sites

Phase 1 (Quantitative): All recruitment of up to 5000 U.S. adults will be performed through the online survey platform Dynata. No participants will be directly recruited at NYULH.

Reasons for Withdrawal or Termination

Participation in this study is voluntary and participants are free to withdraw from the study at any time upon request.

Handling of Participant Withdrawals or Termination

No effort will be made to continue follow-up of withdrawn or terminated participants. Replacement of withdrawn or terminated participants is allowed to reach the enrollment targets.

Premature Termination or Suspension of Study

This study may be temporarily suspended or prematurely terminated if there is sufficient reasonable cause.

Administration of Intervention

Participants will be randomized to watch videos by 1 of 4 speakers about prostate cancer. These videos will have a similar script narrated by a Black doctor, White doctor, Black patient and White patient. The script was created by the study team with input from the stakeholder advisory board. Videos will be filmed with consenting participants by the NYU Videographers. Participants can view the video and survey via the internet (e.g., from a computer or tablet) at any location of their choice (e.g., from their home). This will be done in a single session lasting approximately 15 minutes.

Procedures for Training Interventionalists and Monitoring Intervention Fidelity

The intervention videos will be created through multi-disciplinary collaboration between the study team, stakeholder advisory board and NYU videography experts.

We will attempt to ensure that all participants watch the full video by enabling the survey questions only after the video is complete.

Assessment of Subject Compliance with Study Intervention

Only those participants who complete the entire video and survey will receive compensation (issued by Dynata) and count toward the study. Individuals who do not complete the video and survey in entirety will not be compensated and another participant will be asked to complete the survey instead.

Study Specific Procedures

All participants will be given an extensive baseline questionnaire assessing user characteristics. After completing the baseline questionnaire, participants will be randomized to view one of the four videos. Following exposure to the full video, participants will be asked a series of multiple choice questions including whether they trust the information (Likert scale). These activities will take place online using a device of their choice from a location of their choice, and will be a one-time event of approximately 15 minutes. The survey also includes attention questions/ RECAPCHA to for quality control.

Screening

Dynata will identify members of their survey panel who meet the required demographic criteria (U.S. adults age 40+), who will be sent the IRB-approved recruitment message with the survey link; the survey will contain the embedded study video. There is no chart review or medical record review involved in the study. The initial questions of the survey will include screening questions to confirm eligibility and the survey itself includes quality checks; therefore, to account for losses in sample size at each stage we will recruit additional participants upfront, and cease enrollment once the required analytic sample size is met.

Final Study Visit

The online survey with an embedded video is a one-time activity and there will not be any follow-up contact/visits. Individuals who complete the survey will receive the standard incentive from Dynata.

Concomitant Medications, Treatments, and Procedures

N/A; Concomitant usual medical care that participants may or may not be receiving will not be affected by participation in this study.

Adverse Event Reporting

Adverse events will be reported to the IRB by the NYU PI/research coordinator within 7 business days.

Safety Oversight

It is the responsibility of the Principal Investigator to oversee the safety of the study at his/her site. This safety monitoring will include careful assessment and appropriate reporting of adverse events as noted above.

Statistical Hypotheses

(1) Black adults are more likely to trust online PCa content that is racially concordant than content that lacks Black representation. (2) eHealth literacy and medical mistrust are significantly associated with trust in online PCa content.

Analysis Datasets

The analytic dataset will include enrolled participants from Dynata who complete the survey.

Description of Statistical Methods

This study is a randomized trial comparing the trust of U.S. adults between videos with 4 different speakers about prostate cancer using an online survey. See below for more details on analysis.

Analysis of the Primary Efficacy Endpoint(s)

The primary outcome of this study is the level of trust that Black adults report for videos with a physician versus patient. We will also compare trust between videos with a Black presenter versus a White presenter, and screening versus clinical trials topic using the chi-square test.

Although trust is measured along a Likert scale, to provide conservative estimates and in line with previous studies, our power calculation was performed with trust as a dichotomous variable and assuming a 10% difference in proportions. With a final analytic sample size of 1200 Black adults, we have 92% power to detect a 10% difference in trust based upon the race of the presenter, or the qualifications of the speaker. Adjusting for multiple comparisons using the False Discovery Rate Method (Benjamini-Hochberg), this sample size provides 92% power.

Analysis of the Secondary Endpoint(s)

Secondary analysis will examine the relationship between eHealth literacy (using the published eHEALS) and medical mistrust with trust of the video. All of these measures will be measured on the one-time online survey. On the survey all items are required for completion in order for the survey to be submitted and count toward the final total. Any incomplete surveys/withdrawals will be replaced by Dynata. Therefore, we expect complete data for all covariates.

Safety Analyses

N/A

Adherence and Retention Analyses

The study involves a one-time online survey.

Baseline Descriptive Statistics

All participants will be given an extensive baseline questionnaire assessing user characteristics. Descriptive statistics will be used to tally the results to the baseline questionnaires for the overall study population.

Planned Interim Analysis

N/A

Safety Review

N/A

Sample Size

We will recruit up to 5000 U.S. adults using the nationwide platform Dynata, with an objective of at least 1200 Black adults and 1200 White adults completing the survey (including at least 900 men). This sample size was determined based on our power calculation for the primary outcome of trust (see power calculation information above).

Enrollment/Randomization/Masking Procedures

The survey will randomize a video featuring one of 4 different speakers embedded within a de-identified online survey to be shown to each participant. The survey will not collect any PII or PHI and we will not know who the individual respondents are. The randomization will be done separately stratified by race (separate computerized randomization in Black adults and White adults). Individuals who do not complete the survey will be replaced by Dynata to ensure that the enrollment target is met. The study team will not have any direct contact or access to the study participants, and will be responsible for statistical analysis of the final survey results. No interim analysis is planned.

Quality Assurance and Quality Control

QC procedures will be implemented beginning with the data entry system and data QC checks that will be run on the database will be generated.

Ethical Standard

The investigator will ensure that this study is conducted in full conformity with Regulations for the Protection of Human Subjects of Research codified in 45 CFR Part 46, 21 CFR Part 50, 21 CFR Part 56, and/or the ICH E6.

Institutional Review Board

The protocol, informed consent form(s), recruitment materials, and all participant materials will be submitted to the IRB for review and approval. Approval of both the protocol and the consent form must be obtained before any participant is enrolled. Any amendment to the protocol will require review and approval by the IRB before the changes are implemented to the study. All changes to the consent form will be IRB approved; a determination will be made regarding whether previously consented participants need to be re-consented.

Consent/Assent and Other Informational Documents Provided to Participants

Consent forms describing in detail the study agent, study procedures and risks are given to the participant prior to starting the intervention/administering the study product. The consent document will be shown on the first page of the online survey. Participants must click to indicate consent to initiate the survey.

Consent Procedures and Documentation

A link to the anonymous online survey will be included with the IRB-approved recruitment message and shared by Dynata with potential participants. The recruitment message will include information about the study and eligibility criteria. Persons interested in participating in the study will have the option to click on an embedded link that will take them to an IRB-approved on-screen consent document where they can read about the study and decide whether they would like to fill out the survey. The user also has the option to read about the study and choose not to participate by leaving the webpage. Finally, the user could also ignore the invitation entirely and choose not to click on the link. All subjects who agree to participate in the online survey will be presented with an on-screen consent document which outlines the study purpose and procedures. Participants will have to click through and agree to the electronic consent text before they can be redirected to the anonymous online survey. Participant decision to complete the online survey is completely voluntary and acknowledgement of the on-screen consent document serves as consent. Subjects face minimal risk by participating in this study and can quit the survey and withdraw from the study at any time.

Participant and Data Confidentiality

Information about study subjects will be kept confidential and managed according to the requirements of the Health Insurance Portability and Accountability Act of 1996 (HIPAA).

Funding Source

This study is funded by a Health Disparity Research Award from the Department of Defense.

Costs to the Participant

The participant will not have to pay for research participation.

Participant Reimbursements or Payments

Participants who complete a survey will be reimbursed through Dynata.

Study Leadership

The Steering Committee will govern the conduct of the study. The Steering Committee will be composed of the PI, co-investigators and research staff. Input into the study will be obtained from the stakeholder advisory board.

Conflict of Interest Policy

The independence of this study from any actual or perceived influence, such as by the pharmaceutical industry, is critical. Therefore any actual conflict of interest of persons who have a role in the design, conduct, analysis, publication, or any aspect of this trial will be disclosed and managed. All NYULH investigators will follow the applicable conflict of interest policies.

Supplemental File. Statistical Analysis Plan.

The primary outcomes of this study are the level of trust that Black adults report for videos with a Black presenter versus a White presenter, with a physician versus patient, and for the screening versus clinical trials topic, using the chi-square test.

Although trust is measured along a Likert scale, to provide conservative estimates and in line with previous studies,[152, 158] our power calculation was performed with trust as a dichotomous variable and assuming a 10% difference in proportions. With a final analytic sample size of 1200 Black adults, we have 92% power to detect a 10% difference in trust based upon the race of the presenter, or the qualifications of the speaker (**Table**). Adjusting for multiple comparisons using the False Discovery Rate Method (Benjamini-Hochberg), this sample size provides 92% power. A parallel similar analysis will be performed in White adults based on the same power calculation.

Sample Size	200	300	400	500	600	700	800	900	1000	1200	1500
Not adjusted for multiple comparisons	25%	36%	47%	57%	65%	72%	77%	84%	86%	92%	96%
Adjusted for Multiple Comparisons	15%	27%	39%	50%	60%	68%	74%	82%	85%	92%	96%
