# Supplementary material for: The Effect of Racial Concordance on Patient Trust in Online Videos About Prostate Cancer: A Randomized Clinical Trial
Source: JAMA Netw Open. 2023 Jul 19;6(7):e2324395. doi: 10.1001/jamanetworkopen.2023.24395 (PMC10357333; doi:10.1001/jamanetworkopen.2023.24395)
Supplement: Supplement 2. — eTable 1. Demographics for Black Participants Who Were Randomized to Each of 8 Different Videos eTable 2. Demographics for White Participants Who Were Randomized to Each of 8 Different Videos eTable 3. Multivariable Logistic Regression Model for Trust in Prostate Cancer Videos According to Video Characteristics (Race of Speaker, Qualifications of Speaker and Topic of the Video), Mutually Adjusted, Among 1703 Black Adults Stratified by Health Literacy (Limited or Adequate) eFigure. Flow Chart for Selection of the Study Population [file jamanetwopen-e2324395-s002.pdf]

Supplemental Online Content

Loeb S, Ravenell JE, Gomez SL, et al. The effect of racial concordance on patient trust in online videos about prostate cancer: a randomized clinical trial. *JAMA Netw Open*. 2023;6(7):e2324395. doi:10.1001/jamanetworkopen.2023.24395

eTable 1. Demographics for Black Participants Who Were Randomized to Each of 8 Different Videos

eTable 2. Demographics for White Participants Who Were Randomized to Each of 8 Different Videos

eTable 3. Multivariable Logistic Regression Model for Trust in Prostate Cancer Videos According to Video Characteristics (Race of Speaker, Qualifications of Speaker and Topic of the Video), Mutually Adjusted, Among 1703 Black Adults Stratified by Health Literacy (Limited or Adequate)

eFigure. Flow Chart for Selection of the Study Population

This supplemental material has been provided by the authors to give readers additional information about their work.

© 2023 Loeb S et al. *JAMA Network Open.*

eTable 1. Demographics for Black Participants Who Were Randomized to Each of 8 Different Videos

	Black MD Screening (n=212)	Black MD Clinical Trial (n=214)	Black Patient Screening (n=212)	Black Patient Clinical Trial (n=207)	White MD Screening (n=209)	White MD Clinical Trial (n=218)	White Patient Screening (n=223)	White Patient Clinical Trial (n=208)
Age (mean, SD, range)	56.2 (11.2, 40-85)	54.6 (11.2, 40-81)	55.4 (11.0, 40-80)	55.1 (11.1, 40-80)	55.7 (10.8, 40-82)	55.7 (10.5, 40-81)	55.5 (11.2, 40-91)	56.0 (11.3, 40-87)
Gender (n, %)								
Women	100 (47.2)	97 (45.3)	92 (43.4)	100 (48.3)	95 (45.5)	96 (44.0)	113 (50.7)	109 (52.4)
Men	112 (52.8)	117 (54.7)	120 (56.6)	107 (51.7)	114 (54.6)	122 (56.0)	110 (49.3)	99 (47.6)
Ethnicity (n, %)								
Non-Hispanic	199 (93.9)	198 (92.5)	193 (91.0)	188 (90.8)	191 (91.4)	207 (95.0)	209 (93.7)	197 (94.7)
Hispanic	12 (5.7)	16 (7.5)	18 (8.5)	18 (8.7)	17 (8.1)	11 (5.1)	12 (5.4)	11 (5.3)
Missing	1 (0.5)	0 (0.0)	1 (0.5)	1 (0.5)	1 (0.5)	0 (0.0)	2 (0.9)	0 (0.0)
Education (n, %)								
< High school	2 (0.9)	2 (0.9)	4 (1.9)	2 (1.0)	3 (1.4)	6 (2.8)	2 (0.9)	5 (2.4)
High school	36 (17.0)	34 (15.9)	41 (19.3)	43 (20.8)	36 (17.2)	49 (22.5)	44 (19.7)	45 (21.6)
Some college	57 (26.9)	56 (26.2)	56 (26.4)	61 (29.5)	61 (29.2)	53 (24.3)	66 (29.6)	69 (33.2)
College	75 (35.4)	79 (36.9)	73 (34.4)	54 (26.1)	74 (35.4)	72 (33.0)	65 (29.2)	56 (26.9)
Grad./Profess.	42 (19.8)	43 (20.1)	38 (17.9)	44 (21.3)	34 (16.3)	38 (17.4)	43 (19.3)	32 (15.4)
Other	0 (0.0)	0 (0.0)	0 (0.0)	3 (1.5)	1 (0.5)	0 (0.0)	3 (1.4)	1 (0.5)
Cancer History (n, %)								
None	186 (87.7)	180 (84.1)	181 (85.4)	172 (83.1)	174 (83.3)	194 (89.0)	200 (89.7)	177 (85.1)
Prostate Cancer	20 (9.4)	21 (9.8)	21 (9.9)	24 (11.6)	18 (8.6)	17 (7.8)	15 (6.7)	19 (9.1)
Other Cancer	6 (2.8)	13 (6.1)	10 (4.7)	11 (5.3)	17 (8.1)	7 (3.2)	8 (3.6)	12 (5.8)
Health Literacy (n, %)								
Adequate	68 (32.1)	60 (28.0)	68 (32.1)	69 (33.3)	61 (29.2)	59 (27.1)	71 (31.8)	63 (30.3)
Limited	144 (67.9)	154 (72.0)	144 (67.9)	138 (66.7)	148 (70.8)	159 (72.9)	152 (68.2)	145 (69.7)
E-health Literacy (mean, SD, range)	31.4 (5.9, 8-40)	31.4 (5.9, 8-40)	32.3 (5.4, 8-40)	32.6 (5.3, 8-40)	31.7 (5.3, 8-40)	31.7 (5.6, 8-40)	31.7 (5.2, 10-40)	32.4 (6.4, 8-40)

© 2023 Loeb S et al. *JAMA Network Open.*

Medical Mistrust (mean, SD, range)	13.9 (6.3, 6-30)	14.1 (6.2, 6-30)	13.9 (6.4, 6-30)	13.8 (6.4, 6-30)	13.9 (6.5, 6-30)	13.9 (6.0, 6-30)	14.5 (6.1, 6-30)	13.2 (6.3, 6-30)

© 2023 Loeb S et al. *JAMA Network Open.*

eTable 2. Demographics for White Participants Who Were Randomized to Each of 8 Different Videos

	Black MD Screening (n=148)	Black MD Clinical Trial (n=152)	Black Patient Screening (n=152)	Black Patient Clinical Trial (n=148)	White MD Screening (n=154)	White MD Clinical Trial (n=144)	White Patient Screening (n=149)	White Patient Clinical Trial (n=154)
Age (Mean, SD, range)	63.0 (11.4, 40-83)	63.2 (12.6, 40-86)	63.5 (12.5, 40-87)	62.8 (11.8, 40-85)	62.5 (11.5, 40-83)	62.6 (12.5, 40-88)	62.3 (11.5, 40-85)	64.3 (11.0, 40-91)
Gender (n, %)								
Women	38 (25.7)	42 (27.6)	37 (24.3)	36 (24.3)	38 (24.7)	35 (24.3)	38 (25.5)	37 (24.0)
Men	110 (74.3)	110 (72.4)	115 (75.7)	112 (75.7)	116 (75.3)	109 (75.7)	111 (74.5)	117 (76.0)
Ethnicity (n, %)								
Non-Hispanic	148 (100.0)	152 (100.0)	152 (100.0)	146 (98.7)	152 (98.7)	144 (100.0)	148 (99.3)	153 (99.4)
Hispanic	0 (0.0)	0 (0.0)	0 (0.0)	0 (0.0)	2 (1.3)	0 (0.0)	0 (0.0)	1 (0.7)
Missing	0 (0.0)	0 (0.0)	0 (0.0)	2 (1.4)	0 (0.0)	0 (0.0)	1 (0.7)	0 (0.0)
Education (n, %)								
< High school	5 (3.4)	3 (2.0)	3 (2.0)	1 (0.7)	3 (2.0)	1 (0.7)	2 (1.3)	1 (0.7)
High school	32 (21.6)	23 (15.1)	26 (17.1)	20 (13.5)	30 (19.5)	21 (14.6)	24 (16.1)	27 (17.5)
Some college	42 (28.4)	39 (25.7)	32 (21.1)	38 (25.7)	36 (23.4)	44 (30.6)	51 (34.2)	33 (21.4)
College	43 (29.1)	48 (31.6)	64 (42.1)	55 (37.2)	51 (33.1)	49 (34.0)	50 (33.6)	66 (42.9)
Grad/Profess.	26 (17.6)	39 (25.7)	27 (17.8)	34 (23.0)	33 (21.4)	29 (20.1)	21 (14.1)	27 (17.5)
Other	0 (0.0)	0 (0.0)	0 (0.0)	0 (0.0)	1 (0.7)	0 (0.0)	1 (0.7)	0 (0.0)
Cancer History (n, %)								
None	127 (85.8)	121 (79.6)	126 (82.9)	121 (81.8)	132 (85.7)	126 (87.5)	125 (83.9)	132 (85.7)
Prostate Cancer	6 (4.1)	12 (7.9)	13 (8.6)	14 (9.5)	9 (5.8)	9 (6.3)	8 (5.4)	10 (6.5)
Other Cancer	15 (10.1)	19 (12.5)	13 (8.6)	13 (8.8)	13 (8.4)	9 (6.3)	16 (10.7)	12 (7.8)
Health Literacy (n, %)								
Adequate	55 (37.2)	49 (32.2)	50 (32.9)	52 (35.1)	47 (30.5)	43 (29.9)	51 (34.2)	51 (33.1)
Limited	93 (62.8)	103 (67.8)	102 (67.1)	96 (64.9)	107 (69.5)	101 (70.1)	98 (65.8)	103 (66.9)
E-health Literacy (mean, SD, range)	31.7 (5.5, 15-40)	31.7 (5.5, 15-40)	30.7 (5.9, 9-40)	31.0 (5.2, 10-40)	30.7 (5.9, 8-40)	30.1 (5.7, 16-40)	30.7 (5.6, 10-40)	30.6 (6.5, 8-40)
Medical Mistrust (mean, SD, Range)	10.6 (5.5, 6-28)	9.9 (5.2, 6-30)	9.0 (4.5, 6-26)	10.2 (4.6, 6-25)	9.3 (4.4, 6-27)	9.4 (4.1, 6-21)	10.5 (5.2, 6-27)	9.5 (4.6, 6-25)

© 2023 Loeb S et al. *JAMA Network Open.*

eTable 3. Multivariable Logistic Regression Model for Trust in Prostate Cancer Videos According to Video Characteristics (Race of Speaker, Qualifications of Speaker and Topic of the Video), Mutually Adjusted, Among 1703 Black Adults Stratified by Health Literacy (Limited or Adequate)

**Variables**	**Limited Health Literacy (n = 1184) Adjusted OR (95% CI), p-value**		**Adequate Health Literacy (n = 519) Adjusted OR (95% CI), p-value**	
	Univariable Regression	Multivariable Regression	Univariable Regression	Multivariable Regression
Black vs White (ref.) Speaker	1.38 (1.09 - 1.75), p = 0.008*	1.38 (1.08, 1.75), p = 0.009*	1.82 (1.19, 2.78), p = 0.005*	1.85 (1.20, 2.84), p = 0.005*
Patient vs Doctor (ref.) Speaker	0.75 (0.59, 0.95), p = 0.017*	0.75 (0.59, 0.95), p = 0.016*	0.49 (0.32, 0.75), p = 0.001*	0.48 (0.31, 0.75), p = 0.001*
Clinical Trial vs Screening (ref.) Topic	0.88 (0.69, 1.12), p = 0.30	0.87 (0.69, 1.11), p = 0.27	0.67 (0.44, 1.02), p = 0.06	0.66 (0.43, 1.01), p = 0.06

© 2023 Loeb S et al. *JAMA Network Open.*

eFigure. Flow Chart for Selection of the Study Population

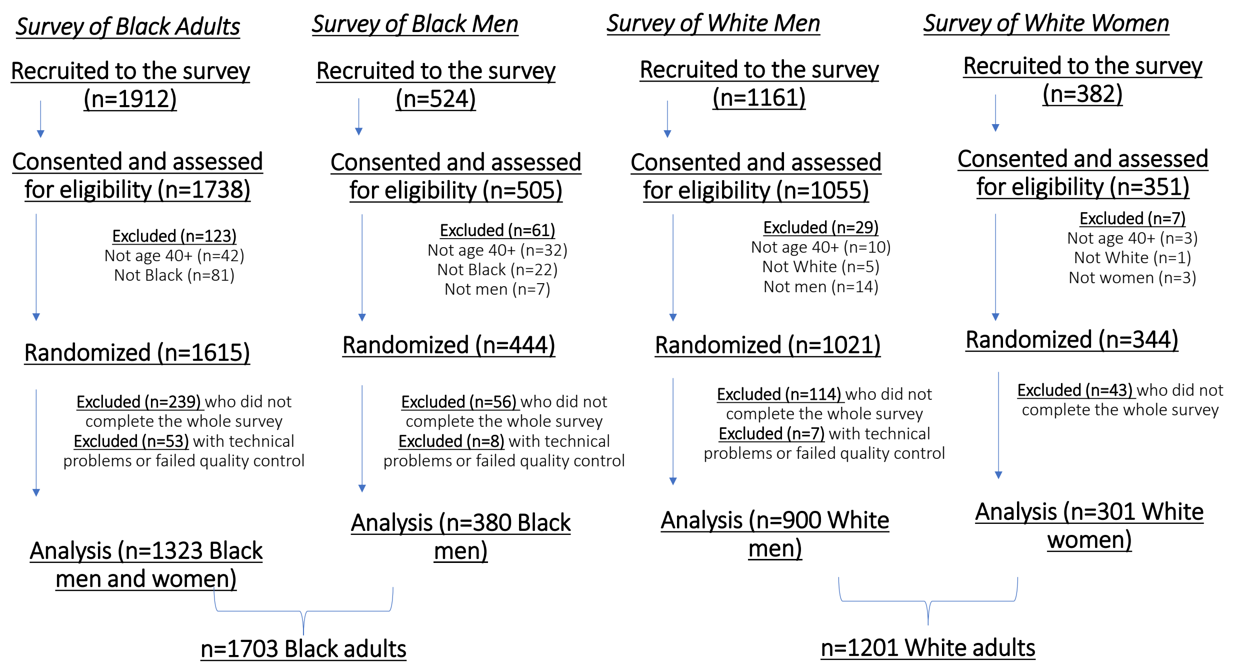

© 2023 Loeb S et al. *JAMA Network Open.*
