# Supplementary material for: The Effect of Racial Concordance on Patient Trust in Online Videos About Prostate Cancer: A Randomized Clinical Trial
Source: JAMA Netw Open. 2023 Jul 19;6(7):e2324395. doi: 10.1001/jamanetworkopen.2023.24395 (PMC10357333; doi:10.1001/jamanetworkopen.2023.24395)
Supplement: Supplement 3. — Data Sharing Statement [file jamanetwopen-e2324395-s003.pdf]

Data Sharing Statement

Loeb. The Effect of Racial Concordance on Patient Trust in Online Videos about Prostate Cancer. *JAMA Netw Open*. Published July 19, 2023.
doi:10.1001/jamanetworkopen.2023.24395

Data
Data available: Yes
Data types: Deidentified participant data, Other (please specify)
Additional Information: Data will be available only for health/medical/biomedical research purposes.
How to access data: Interested investigators will be required to submit a formal letter of intent outlining research aims, rationale, and approach. Furthermore, documentation of local IRB approval, including a description of type of review, should be submitted with the data request.
When available: With publication

Supporting Documents
Document types: None

Additional Information
Who can access the data: Interested investigators will be required to submit a formal letter of intent outlining research aims, rationale, and approach. Documentation of local IRB approval, including a description of type of review, should be submitted with the data request.
Types of analyses: A Data Use Agreement must be signed by the Institutions involved before any data are released. The nature and scope of the project will be described in a written collaborative agreement signed by the outside collaborator, the Principal Investigators, and a representative from each investigator's institution. Use of the data will be for the specific approved project. If further research develops from the original project, approval for that research must be obtained by the procedures described here.
Mechanisms of data availability: Data will be made available after the Data Use Agreement is signed.
Any additional restrictions: N/A
